# Creating conditions for Canadian aboriginal health equity: the promise of healthy public policy

**DOI:** 10.1186/s40985-016-0016-5

**Published:** 2016-07-20

**Authors:** Chantelle A. M. Richmond, Catherine Cook

**Affiliations:** 1 Department of Geography, Western University, London, Ontario Canada; 2 Population and Aboriginal Health, Winnipeg Health Region, Winnipeg, Manitoba Canada; 3 Institute of Indigenous Health, University of Manitoba, Winnipeg, Manitoba Canada

**Keywords:** Aboriginal health, Healthy public policy, Health equity, Colonialism, Racism, Self-determination

## Abstract

In the Canadian context, the persistence and growth of Aboriginal health and social inequity signals that we are at a critical public health policy juncture; current policy reflects an historic relationship between Aboriginal people and Canada that fails the contemporary health needs of Canada’s Aboriginal peoples. In this review, we highlight the need for healthy public policy that recognizes and prioritizes the rights of Canada’s Aboriginal people to achieve health equity. Drawing from a structural approach, we examine the historical scope and comprehensive breadth of the Indian Act in shaping modern Aboriginal health and social inequities. Canada’s failure to implement a national public policy for Aboriginal health reflects the proliferation of racism in modern day Canada, and a distinctly lacking political will at the federal level. Despite these structural challenges, there is great promise in community self-determination in health care and the role of community-led research as advocacy for policy reform. In our conclusion, we turn to the Report on the Truth and Reconciliation Commission of Canada (2015) and draw upon the concept of reconciliation as a fundamental precursor for Aboriginal health equity. The burden of systemic change needed to promote healthy public policy cannot be carried by any single group of advocates; it is a shared responsibility that will require the collaboration and integration of various actors and knowledges.

## Background

The concept of healthy public policy was originally introduced in the Ottawa Charter for Health Promotion [1] (1986), as a tool to create the sorts of supportive environments that will enable people to live healthy lives by putting health on the policy agenda in all sectors, and at all levels. The Ottawa Charter has become a powerful addition to public health theory and practice globally. It was informed by the 1974 Lalonde Report [2], wherein the concept of the ‘health field’ was first proposed to include four major elements: genetics, environment, lifestyle, and medical care. The Lalonde Report was conceptually important as it introduced the idea that there are determinants, distinct from the health care system, that affect the health of individuals and populations. Healthy public policy recognizes that the health of a population requires investment and coordination on a *whole range* of economic, social, environmental and political forces. There is also recognition that in creating the conditions for equitable access to health services among vulnerable populations, such as that of Indigenous peoples, particular attention must be paid to their unique cultural contexts and histories [3].

In spite of the important conceptual development of healthy public policy at the global level, there is a distinct absence of Canadian public policy supporting Aboriginal^1^ health and well-being at the national and provincial levels [4, 5]. While the last half century has witnessed significant gains in life expectancy for Canada’s Aboriginal population and a considerable reduction in infant mortality, we see several troubling patterns of health including a high burden of chronic and infectious disease [6] paired with severely inadequate approaches for addressing the social determinants of Aboriginal health [7]. The persistence and growth of Aboriginal health and social inequity [8] signals that we are at a critical public health policy juncture with regard to the health of Canada’s Aboriginal peoples [9].

Today in Canada, the only active national-level legislation specific to First Nations people remains the Indian Act of 1876 [10], which gave responsibility of health and health care for First Nations to the federal government, while for the general population, health was primarily a provincial responsibility. Originally introduced with a broader goal of assimilation, the Indian Act was developed and implemented under the assumption that the Aboriginal population was inferior, unequal, and uncivilized [11]. Nearly 140 years after its introduction, the strategies and multiple amendments imposed to enforce the Indian Act – including the establishment of the Indian Residential Schools – have been labelled “cultural genocide” [12]. The effects of the Indian Act are pervasive in all modern health, social, economic and political indicators of Aboriginal well-being [7–9], and many claim that the Indian Act has served to perpetuate health inequity, as well as racism and gender discrimination, which are themselves, important determinants of health [13, 14]. Perhaps more troubling however, is the way the Indian Act has shaped the public purview of the Aboriginal population as a “sick and defenseless,” burden to Canadian society, and simultaneously proliferated the need for continued federal control of the Aboriginal population through programs and systems that remain significantly underfunded when compared to similar programs for non-Aboriginal Canadians [15, 16]. This perception has been perpetuated in educational curricula nationwide and in the often-negative media coverage of events or situations involving Aboriginal people that further reinforce racist or negative stereotypes [14, 17].

In this review, we draw from a structural approach to contextualize the ways the historical structure of colonialism – enacted through the Indian Act – frames contemporary Canadian Aboriginal health inequity [18]. A structural approach to health focuses its attention on understanding the complex relationship between the organizational structure of a particular society – including the morals and ethics upon which this structure is founded –and its related impact on health and well-being [19]. This paper opens with an examination of the Indian Act and the comprehensive breadth of its historical scope in shaping the modern health and social status of Aboriginal Canada [7, 13, 14, 18]. We then move into a discussion of both the challenge and promise of healthy public policy for Aboriginal Canada. In our conclusion, we turn to the Report on the Truth and Reconciliation Commission of Canada (2015) and draw upon the concept of reconciliation as a precursor for building the sort of healthy public policy that may lead to Aboriginal health equity^2^.

## The Indian Act

Prior to colonization, Indigenous societies could be described as subsistence cultures, meaning that their diet, daily nourishment and medicines were provided by the resources of their local ecosystems. This reliance on the ecosystem nurtured a deep cognitive, spiritual and physical relatedness to their lands and resources [20] that was maintained through local knowledge systems and formed the cornerstone of Indigenous way of life [21]. At the most basic level, it was these distinct knowledge systems – practiced by individuals, families and communities over time immemorial – that seeded the roots for Indigenous societies to flourish in their social, political, cultural, economic and spiritual systems.

During the colonization of Canada, clashing philosophical understandings about development, religion, and land ownership – among many other ways of knowing the world – between Indigenous people and new settlers created a number of challenges for the developmental agenda of the new nation [22, 23]. As early as 1867, recognition of the need to manage what was later termed the “Indian Problem.” As noted, the Indian Act gave the federal government of Canada constitutional responsibility for ‘Indian Affairs,’ under Section 91.24 of the Constitution Act. This mandated the federal government with the unilateral responsibility for all matters relating to “Indians and Indian lands.” From 1871 to 1921, several treaties were signed between Indian people and the Crown; in exchange for land for new settlers and the nation state, these treaties established the rights of Indians to a number of provisions including such things as reserve lands, farming equipment and animals, annual payments, ammunition, clothing and certain rights to hunt and fish. In spite of the coordination of affairs at the provincial level for the general Canadian population (e.g., health, education, energy, labour), as early as the mid-1800’s, the delivery of a broad range of services for First Nations – including health services – have been the jurisdiction of the federal government [17, 24–26], thus creating a jurisdictional ambiguity over Indian health that remains today [27].

In 1876 the Indian Act was legislated with the fundamental goal of ‘civilizing the Indians,’ and created under the assumption that Aboriginal people and their ways of living were inferior, unequal and uncivilized. The Indian Act included provisions that extended across the social, cultural, economic, political, gender and even spiritual dimensions of Indian life. It imposed religious and education systems, formal ownership of lands, and permanent settlement on lands reserved for Indians, foreign systems of government, mandated participation in foreign systems of wage labour and employment, and encouraged Aboriginal people to relinquish their Aboriginal status and treaty rights [15, 25, 26]. Many of these provisions were diametrically opposed to pre-existing principles of governance, and moral and social order that were in place in Indigenous communities prior to colonization [28]. For example, the Indian Act held provisions that promoted gender bias toward men [13]. An Indian woman’s status – and therefore her access to her Aboriginal and treaty rights – was dependent fully on the legal status of her husband. According to Section 12 (1)(b) of the Indian Act, “a woman who married a person who is not an Indian… [is] not entitled to be registered.” If a status Indian woman married a non-Indian man, her Indian status would be terminated and she would lose treaty benefits, health benefits, the right to live on her reserve, the right to inherit her family property, and even the right to be buried on the reserve with her ancestors. Paradoxically, if an Indian man married a non-status woman, he would retain his rights and his new wife would gain status and associated rights and benefits. This gender bias has had devastating consequences for families and communities across Canada. Recent amendments to address gender inequality in the Indian Act are ongoing, both through Bill C-31 (1985) and the McIvor Decision (2012).

The measures enacted through the Indian Act were part of a coherent set of structures put in place to eliminate Aboriginal people as distinct peoples and to assimilate them into the Canadian mainstream against their will [12]. Deputy Minister of Indian Affairs Duncan Campbell Scott outlined the goals of that policy in 1920, when he told a parliamentary committee that “our objective is to continue until there is not a single Indian in Canada that has not been absorbed into the body politic” [12] (p.3). These colonial structures set the stage for a debilitating, systemic public policy that continues, in the modern day, to powerfully shape patterns of Aboriginal health, social inequity and access to health care and other services. Historically, the systems of care for Aboriginal people (health, education, child welfare, justice, economic development) were developed, and continues to provide services, based on a foundation of racial discrimination, colonialism and a lack of recognition of the self-determination of Aboriginal peoples and governance in communities [14, 15, 17].

## Contemporary patterns of aboriginal health

Under Section 35(2) of the Constitution Act, 1982 [29, 30], the Aboriginal Population of Canada is composed of three legally identified groups: Indian, Inuit and Métis. In 2011 1.4 million Canadians reported Aboriginal *identity* (that is, 697,510 First Nations, 418,380 Métis, and 59,115 Inuit) [31]. Aboriginal Canada’s demographic profile^3^ reflects a young, quickly growing population, characterized by high birth rate and low life expectancy. Canada’s Aboriginal population is growing faster than the general population, increasing by 20.1 % from 2006 to 2011 (compared with 5.2 % growth rate in the non-Aboriginal population). This is due to a higher fertility rate among Aboriginal women than among other Canadian women. Of the three Aboriginal groups (First Nations, Métis, Inuit), First Nations had the largest population growth, with an increase of 22.9 % between 2006 and 2011 [6]. First Nations women are having babies at significantly younger ages; over half of First Nations women who gave birth in 1999 were less than 25 years old [33]. Secondly, while life expectancy is increasing across all Aboriginal groups, it still lower than the non-Aboriginal population (68.9 for Aboriginal males and 76.6 for Aboriginal women *versus* 78 among non-Aboriginal males and 81 for non-Aboriginal women). Within the Aboriginal population however, there is quite a bit of variation. In 2010, Inuit men had the lowest life expectancy at 64 years, followed by First Nation’s men at 73–74 years of age [34].

Although the national infant mortality rate for the First Nations population in Canada as a whole remains unavailable [35, 36], studies in specific regions indicate a significant disparity in infant mortality between Aboriginal and non-Aboriginal populations [37]. Luo et al. [38] report infant mortality rates that are twice as high among First Nations than non-First Nations in British Columbia, with greater disparity in rural areas (13.8 versus 6.1 deaths per 1000 live births in rural areas; 12.7 versus 6.1 deaths per 1000 live births in urban areas). Results from Manitoba reveal the infant mortality rate for First Nations was twice that of non-First Nations in Manitoba (9.8 versus 5.0 per 1000, respectively) [39].

The overall leading causes of Aboriginal mortality are: injury and poisoning [40, 41], circulatory disease [42], cancer [43], and respiratory disease [44], Chronic diseases also disproportionately affect Aboriginal populations in Canada [45], the most significant of which is diabetes [46, 47].^.^ Rates of diabetes among First Nations, Inuit and Métis are 3 to 5 times the national average, with rates higher among women and highest among those living on-reserve [48]. In terms of morbidity, Aboriginal people also experience a disproportionate burden of infectious disease, including pertussis, chlamydia, hepatitis A, shillegosis, and tuberculosis [49]. HIV/AIDS diagnoses in the Aboriginal population are also on the rise [50]. In 2011, Aboriginal peoples accounted for 12.2 % of new HIV infections and 18.8 % of reported AIDS cases [51].

The most common cause of death among ages 1 – 44 is injury and poisoning. Among children under ten, these deaths were primarily unintentional. Among youth and adults up to age 44, suicide and self-injury were the leading causes of death [41]. The suicide rate for First Nations males aged 15–24 years is 126 per 100,000 compared to 24 per 100,000 for non-Aboriginal males. The First Nations female suicide rate is 35 per 100,000 compared to five per 100,000 for non-Aboriginal females [52]. For Inuit, these numbers are comparable. During the period 1994–1998 to 2004–2008 the suicide rate for girls and young women (aged 1–19) in Inuit populations were more than 20 times the rate for the non-Aboriginal Canadian population at 40 deaths/100,000 (person-years at risk: PYR) compared to two deaths/100,000 PYR in the general population. For Inuit boys and young men (aged 1–19) in the same study the suicide rates were 101.6 deaths/100,000 PYR during 2004–2008 compared with 4.2/100,000 PYR for the rest of Canada’s population [53]. With respect to suicide, all First Nations and Inuit groups up to age 65 are at increased risk, in comparison with the Canadian population. While males are at a higher risk of both attempted and completed suicides than females, the greatest disparity with the non-Aboriginal rates is for females aged 15–24 and 25–29, for whom the rates of suicide are eight and five times non-Aboriginal rates [54, 55]. For those ages 45 and older, circulatory disease was the most common cause of death [6, 42].

## The social determinants of health

Beyond traditional health measures, Aboriginal peoples also endure a disproportionate burden of disparity related to workforce participation, low income, education, and sub-standard living conditions. In 2005–06, the average rate of [welfare] dependency on reserve was seven times higher than the national rate (36 % compared to 5.5 %) [56]. Comparisons between on and off reserve Indians and the non-Aboriginal Canadian population indicates that Aboriginal household incomes are substantively lower than their non-Aboriginal counterparts [55]. In 2009 the off-reserve unemployment rate was 13.9 %, compared to 8.1 % in the general population [57]. The on-reserve Aboriginal unemployment rate in 2006 was considerably higher at 23.1 % [58]. In 2005, the average income for the total on and off reserve Indian population aged 25 to 54 was $22,366, substantially lower than the reference non-Aboriginal population income of $33,394 [59]. The income disparity between Aboriginal and non-Aboriginal populations is greatest for on-reserve First Nations with a median income of just over $14,000. While First Nations children are staying in school longer than in the recent past, there remains an across-the-board lag in completion rates at all levels of education when compared to the non-Aboriginal population [60]. According to the 2012 Aboriginal Peoples Survey, 72 % of First Nations people aged 18 to 44 living off reserve had completed the requirements for a high school diploma or equivalent, compared with 89 % of non-Aboriginal peoples aged 18–44 in 2013. Inuit people’s educational attainment is either lower than or comparable to First Nation’s rates, with proportionately fewer Inuit attaining a university degree [61].

In terms of living conditions, inadequate and insufficient housing remains a critical problem across Aboriginal Canada. In the rural and remote context, many Aboriginal households suffer a lack of basic sanitary infrastructure. In 2006, for example, First Nations and Inuit households were three and four times as likely to live in a dwelling in need of major repairs, respectively. and almost four times as likely as non-Aboriginal people to live in a crowded dwelling [62]. Anecdotal evidence suggests that if family members did not open their homes to those in need, the issue of ‘overcrowding’ would be identified as an issue of homelessness on-reserve and in Metis communities. ‘Overcrowding’ and ‘homelessness’ are often considered synonymous in Aboriginal communities despite the persistent descriptor of ‘overcrowding’ by government and public reports of on-reserve situational realities.

The poor and often crowded condition of dwellings is especially common on reserves, where almost 20 % of First Nation communities in Canada are under drinking water advisories [63]. All Aboriginal communities experience increased safety risk when it comes to drinking water. According to recent reports, First Nations experience rates of illness caused by unsafe drinking water at a rate 26 times higher than the national average [64]. The higher incidence rate of waterborne illnesses and large number of communities living under drinking water advisories illustrate the seriousness of water quality issues and safe drinking water challenges for First Nations [65, 66], and not only in remote or isolated communities as one might suspect.

## Challenges to Healthy Public Policy for Aboriginal Canadians

In the modern context, the health and social inequities borne by Aboriginal Canada are rooted fundamentally in their historical position within the Canadian social system [18, 67]. In spite of treaty and other Aboriginal rights protected in the Canadian Constitution, including access to health care, contemporary Aboriginal policy remains characterized by jurisdictional ambiguity, wherein there is today significantly lacking clarity about both the federal and provincial government’s level of health service delivery and financial responsibilities to First Nation and Inuit communities [17]. Canada’s current Aboriginal legislation and health policy framework is rooted in the historic relationship between Canada and Indians living on reserve, and thus does not adequately address the health care needs of the Métis or First Nations and Inuit people who either are not registered or do not live on reserve or in their traditional territory [68].

Contemporary Aboriginal health policy also demonstrates widespread neglect [16], and a distinctly lacking political will to improve access to health and health care. For example, the Auditor Generals’ *Report on Access to Health Services in Remote Communities* [69] identified substantial concerns about the quality of care in remote First Nation communities, citing a number of critical issues that compromise both provision and management of health care including: the inability or unwillingness of government to ensure the competency of service providers; low perceived safety of health care facilities, untimely record keeping with respect to non-insured health benefits, and poor community consultation. Geography intersects in important ways with other social determinants of health to influence the ways access to health care is structured and quality compromised [70–72]. In the Manitoba context for example, Martens et al. [73] identified a significantly higher burden of illness for southern First Nation communities compared with those in the northern regions of the province. While a critical component of the difference reflects adherence to a traditional way of life in northern regions, this disparity also illustrates differential access to health services, in particular that related to perceptions about culturally unsafe health care environments [74, 75] and exposure to marginalization and poverty in the southern regions. This is a trend seen not only in Manitoba, but nation-wide [76]. Combined, the poor coordination of health services, lacking access to quality care, and wide geographic variation of communities have reduced the ability of First Nations and Inuit people to access their constitutionally protected right to health care [77, 78].

## Political will

In Canadian history, there are few instances where the political will in Canada has mandated the health and well-being of the Aboriginal community. However two key efforts to establish coordinated processes to address the health issues and disparities of Aboriginal people and their health and health care systems are worth noting: the *Romanow Report on Canadian Health Care* [79] and the Kelowna Accord [80]. The *Romanow Report*, written by a committee led by Roy Romanow, examined the future of health care in Canada and identified the structural changes need to improve the health care. The report identified Aboriginal health inequity to be rooted in two key issues: a general mismanagement of funding; and a poorly established system to provide care [79]. In response to these shortcomings, Romanow recommended significant restructuring of Aboriginal health care, stating that all levels of government must come together to address Aboriginal health inequities of Aboriginal peoples, including at the community level.

Based on a series of agreements between the Government of Canada, First Ministers of the Provinces, Territorial Leaders, and the leaders of five national aboriginal organizations in Canada, the 2005 Kelowna Accord sought to improve the education, employment, and living conditions for Aboriginal peoples through the dedication of an unprecedented $5 billion. In the context of improving Aboriginal health, targets were established to reduce infant mortality, youth suicide, childhood obesity and diabetes by 20 % in five years, and 50 % in 10 years. Targets were also set to double the number of Aboriginal health professionals in 10 years to 300 physicians and 2400 nurses. Following the Kelowna Accord, hopes were high within the Aboriginal community and policy and decision makers as the Government of Canada had pledged to continue to work inclusively in the development of a policy framework to implement the targets of the Kelowna Accord. Action plans were limited, however. While the Kelowna Accord was endorsed by then Prime Minister Paul Martin, it was never endorsed by his successor, Prime Minister Stephen Harper. Instead, the Health Council of Canada was established as an entity that would oversee the progress of government in achieving the goals of the First Ministers Kelowna Accord for Canadians. Annual Progress Reports with identified targeted were expected to report on the health status of Canadians and a report card on the performance of governments in the federal and provincial domains. When the federal government made the decision not to renew the First Ministers Health Accord, the Health Council of Canada was dismantled, with accountability for some of the mandate reassigned to alternative national organizations that could continue the work, although not in the same comprehensive approach applied by the Health Council. While these examples illustrate the fundamental need and dedication at the provincial level for health policy reform on Aboriginal health and health care, the current federal government’s failure to implement action plans based on these recommendations demonstrates their lacking political will to make health equity a reality for all.

## Racism and sexism in health care

There is a growing body of literature that highlights the impacts of racism on the health and health status of a population, in addition to the racial attitudes and practices that evolve through institutional approaches to system development [81]. Canada is one of the only nations in the world that continues to use legislation to limit access to services and benefits for Aboriginal peoples on the basis of a descent criterion [14]. (p.9) In many Aboriginal communities across Canada, the gender bias of the Indian Act has translated into such egregious proportions where, up until recently, it was possible for male siblings in a biologic family to have legal Aboriginal status while their female siblings did not. This very unequal situation reinforces the conclusion that the persistent efforts and effects of the Canadian government continue to support marginalization of Aboriginal peoples through active legislation like the Indian Act. Race based legislation such as the Indian Act has resulted in the development of institutions or systems that have allowed the entrenchment of racism into the policies and procedures of a suite of organizational structures affecting the daily life and wellbeing of Aboriginal people, including education, health care, justice, economic development, governance and economic development. The literature contains examples of interventions that can address these challenges, including early public education, cultural competence training, and increasing the number of Aboriginal people working in health care settings [7, 12, 14, 81, 82]. For example, part of the efforts to educate medical and nursing students early in their training has been supported through efforts at the academic level, as well as strategies at the provincial and regional health authority levels [82, 83]. The public policy challenges posed by racism are deeply rooted in not only our health care systems, but across multiple domains including communities, governments, places of worship, schools, and workplaces [11], thereby necessitating a public re-education about Aboriginal history and place in Canada. The creation of healthy public policy will rely on an integrated effort across many sectors of public life; as we build the base from which to create such a policy, it will be important for Canadians to understand and appreciate the historic roots of Aboriginal inequity.

## Promises for the creation of healthy public policy

The active role and responsibility of the local community into provision, coordination and delivery of health care and services is an area that the current national policy completely ignores. However, the Adelaide conference on Health Promotion (1988) indicates that a healthy public policy should recognize the unique culture of Indigenous peoples, including acknowledging their inherent right to be self-determining and the Indigenous knowledges they hold, as a fundamental means through which to create the conditions for equal access to health and health care. And indeed, there is good evidence to suggest that that creating supportive healthcare environments that foster Indigenous people’s unique cultures, rights and perspectives will bring about health improvements [74, 83, 84].

## Aboriginal self-determination in health care

Nowhere in Canada is Aboriginal self-determination in health care demonstrated better than in the province of British Columbia (BC). The First Nation Health Authority (FNHA), established in 2011, is the first province-wide health authority of its kind in Canada. With a greater vision of reforming the way health care is delivered to BC’s First Nations, the FNHA has taken ground-breaking steps to meet its mandate, including assuming the delivery and coordination of programs, services, and responsibilities that have historically been handled by the federal government. In an unparalleled process involving a tripartite governance framework including BC First Nations, the Province of BC, and the Government of Canada (First Nations and Inuit Health Branch (FNIHB), the FNHA seeks to address service gaps through partnerships that will foster closer collaboration, and health systems innovation to reform the way health care is delivered to BC First Nations. Perhaps most significantly, at the core of the FNHA strategy for improved health and health care is the placement of the grassroots community. Since 2008, BC First Nations have been involved in an unprecedented process of community engagement to guide the work and set the vision for BC’s First Nations health governance. The result has been the “*The 7 Directives*,” which describes the fundamental standards and instructions for the new health governance relationship. While we could find no scholarly studies measuring the impact of the newly established FNHA on population health indicators, a recent exploration by Lavoie et al. [85] revealed concern over the lacking engagement of urban First Nations in these self-government discussions. As Lavoie et al. [85] argue, the substantive basis of the problem lies in the Canadian national conceptualization of self-government, which refers to First Nations communities and historic land bases. The current federal jurisdictional structure necessitates that the FNIHB obligations are transferred to the FNHA, which requires the engagement of First Nations communities and effectively marginalizes First Nations who do not live on reserve or who are detached from their home communities.

## Research as advocacy for healthy public policy

As reflected in the development of the FNHA, wherein the community’s knowledge and preferences have played a significant, guiding role in the restructuring of the health care system, the development of healthy public policy in the Canadian Aboriginal context will be greatly informed through uptake of community-based research approaches. Participation in community based research represents an active means by which Indigenous communities themselves can participate in, and shape research that will have direct policy influence in their own lives [86, 87]. Community-based research is a collaborative approach to research which is critical for ensuring benefits for both researchers and the researched, including the ability to share in leadership, decision-making, capacity-building and other knowledge and benefits that result from the process [88, 89]. This includes – and perhaps most significantly – demonstrates relevance for local people, and use of this research as a tool for self-determination among Indigenous communities [87, 90].

In Canada today, we are seeing the development and uptake of Indigenous research approaches that are nurtured through community needs and visions, and often led by Indigenous academics and communities themselves [91]. This hopeful new way of doing research is being initiated through the collaborative synergies of government, academia and Aboriginal communities [92]. By privileging the voices of those on the ground level, the promise of these partnership-based projects are rooted in research approaches that empower communities to meet their goals of self-determination [93]. Bio-medical interventions resulting from research are important, and critical for establishing and monitoring the burden of disease experienced by Indigenous populations. However, the need to recognize, measure and apply the principles of self-governance as a fundamental determinant of community health cannot be understated [94]. With heavy emphasis on partnership and collaboration, these research approaches serve as important mechanisms by which to enable meaningful Indigenous participation – and most importantly to integrate their unique knowledges, histories and perspectives – in the creation of healthy public policy.

## Conclusion

In Canada, we are at a critical juncture with regard to healthy public policy. The persistence, and in some cases widening, of the health inequality gap between Aboriginal and non-Aboriginal people highlights the need to critically evaluate the issues underpinning Aboriginal health inequity, including the important role of federal policy. It would be careless to assume that colonialism in Canada has ended. Less than a decade ago, Canada was one of four Western nations (along with the United States, New Zealand, and Australia) who voted against the adoption of the *United Nations Declaration on the Rights of Indigenous Peoples* (*UNDRIP*). Though Canada has since adopted *UNDRIP*, the nation’s initial resistance to the Declaration demonstrates its universal failure to acknowledge both the human rights and inherent rights of its Indigenous peoples. At the same time, the failure to support the Kelowna Accord and the elimination of support for advocates of Aboriginal health policy such as the Health Council of Canada – and a range of other national Aboriginal health organizations in the past several years [95] – would indicate that the federal government retains a vested interest in supporting the highly inequitable Indian Act [12, 14]. There remains a fundamental advantage for government in ensuring that the jurisdictional ambiguity established through this approach to public policy continues to exist. Within this context, there is little clarity on land and treaty rights and the federal government maintains that their delivery of service to First Nations and Inuit people is a policy decision, not a legislative directive [17]. Without national healthy public policy on Aboriginal health, there will be no accountability in addressing the issue of youth suicide, Murdered and Missing Indigenous Women and Girls, and other highly inequitable Aboriginal-specific situations. Without healthy public policy in place – one that including targets, action plans, and means of evaluation – the government has no responsibility to act on Aboriginal issues throughout the continuum of the social determinants of health and no accountability for the poor quality of Aboriginal health care [96].

However, there are reasons to be optimistic. We write this paper at an unprecedented time in Canadian history. In June 2015, Justice Murray Sinclair – First Nation lawyer and Chair of the Truth and Reconciliation Commission (TRC) – and his co-commissioners released the findings and calls to action of the TRC, a five-year long undertaking to hear the truth about the various impacts of the Indian Residential Schools through interviews with former students and their families, as well as staff of the Indian Residential Schools. These testimonies revealed not only the horrific abuses suffered by students of the Indian Residential schools, but of the powerful way these abuses were shared inter-generationally by students to their families and subsequent generations. In this report however, Justice Sinclair indicated that the wounds inflicted from the Indian Residential Schools are not specific only to the Aboriginal population, but rather that the whole of the Canadian population has suffered as a result of the shameful Indian policy from which the Indian Residential Schools were created. At the national level, Canada has suffered the tragic break-down of relationship between Aboriginal and non-Aboriginal Canadians, fuelled mainly by the persistence of racism and other forms of discrimination toward Aboriginal people. The persistent support of the Indian Act reinforces the assumption that the Indigenous People of Canada are not worthy of respect, recognition or equity in access to quality service in all public service systems. In his Report, Justice Sinclair identified the process of reconciliation as critical for the healing of Residential School Survivors, and fundamental for forging a new healthy relationship between Aboriginal and non-Aboriginal Canada:

“To the Commission, reconciliation is about establishing and maintaining a mutually respectful relationship between Aboriginal and non-Aboriginal peoples in this country....In order for that to happen, there has to be awareness of the past.... Without truth, justice, and healing, there can be no genuine reconciliation. Reconciliation is not about “closing a sad chapter of Canada’s past,” but about opening new healing pathways of reconciliation that are forged in truth and justice.” (Sinclair, Truth and Reconciliation Commission, 2015: 10)

As a mechanism for achieving reconciliation at the national level, and across the many dimensions of Canadian and Aboriginal life that have been affected by the Indian Act and its comprehensive policies, Justice Sinclair and his co-commissioners identified the principles set out in the *UNDRIP* as an important starting place. However, just as this reconciliation process will take time to unfold, we recognize that the creation of healthy public policy will require a significant shift in philosophy, a reorientation of public attitudes, a commitment by the federal government in acknowledging the rights of Indigenous Canadians and a good deal of empathy.

We call on governance, health administrators, health care professionals, academic communities and Indigenous communities to continue to create the sorts of evidence that can be used to advocate for transformation at the policy level. We appreciate that the burden of systemic change cannot be carried by any single group of advocates. But we do know that it is in the educational institutions that academics and educators can draw on the most important of tools in their possession – public education – to inspire, inform and educate the next wave of Canadian voters, public policy makers, researchers, and community activities of the need to lobby for a more equitable Canada, one that prioritizes the health and well-being of *all* its citizens.

## Footnotes

1 Aboriginal, First Nation, Inuit and Métis are all terms recognized in the Constitution Act of Canada 1982, Section 35, 2 and are used in this paper to describe the Indigenous Peoples of Canada and their descendants.

2 A full analysis and review of policy specific to Aboriginal health is beyond the scope of this paper, however, we will highlight some key examples that illustrate the persistent lack of commitment to partnering with Aboriginal groups to achieve equitable status in all determinants of health.

3 The available health and social statistics on Aboriginal conditions are not without their limitations, and the reader must be cautioned about these data issues. There are many ways of defining the Aboriginal population, and it is this inconsistency in definition which can result in estimates that vary with respect to the size of the population, and its health and social conditions. For instance, Canada’s Census data are based on the definitions of ethnic origin (ancestry), Aboriginal Identity, Registered Indian, and Band membership. This may be problematic for Aboriginal people who do not self-identify their Aboriginal status or identity on the Census, and further challenging among those Aboriginal peoples who do not respond to the Census at all. These issues may be further complicated as certain segments are over-represented in the available literatures on Aboriginal health and social conditions. For instance, a Medline search of 254 journal articles published during 1992–2001 found that the majority of research on Canada’s Aboriginal population does not reflect the demographic composition of Aboriginal people in Canada, as there is severe under-representation of Métis, urban Aboriginal people, and First Nations people not living on reserves and over-representation of the Inuit [32]. In terms of data representativeness, we must therefore be cautious about misrepresenting statistics to groups who may not, in fact, be captured in these estimates. In the Canadian estimates, First Nations (specifically those living on-reserve) and Inuit populations have been well represented in national level data, while the Métis population have not. The health and social statistics provided in this literature review, for the most part, reflect that of on-reserve Indians and Inuit living in Arctic areas.
